# Comparison of bupivacaine and ropivacaine in combination with fentanyl used for walking epidural anesthesia in labor

**DOI:** 10.4274/tjod.87404

**Published:** 2017-09-30

**Authors:** Şükrü Gündüz, Serenat Eriş Yalçın, Gökhan Karakoç, Mehmet Özgür Akkurt, Yakup Yalçın, And Yavuz

**Affiliations:** 1 University of Health Sciences, Kartal Dr. Lütfi Kırdar Training and Research Hospital, Clinic of Anesthesiology, İstanbul, Turkey; 2 Süleyman Demirel University Faculty of Medicine, Department of Obstetrics and Gynecology, Division of Perinatology, Isparta, Turkey; 3 University of Health Sciences, Ankara Etlik Zübeyde Hanım Women’s Health Training and Research Hospital, Clinic of Perinatology, Ankara, Turkey; 4 Isparta City Hospital, Clinic of Gynecologic Oncology, Isparta, Turkey

**Keywords:** bupivacaine, epidural, fentanyl, labor, ropivacaine

## Abstract

**Objective:**

Effective pain relief during labor is essential to reduce maternal and perinatal morbidity arising due to pain-induced maternal sympathetic activation, and to avoid unnecessary cesarean sections performed due to maternal anxiety. Walking epidural analgesia on labor reveals lower pain scores, leading to higher maternal satisfaction with better cardiovascular and pulmonary physiology. Despite the extensive use and relative safety of bupivacaine, newer drugs such as ropivacaine have been developed as alternative agents to decrease the risk for cardiac and central nervous system toxicity.

**Materials and Methods:**

One hundred women who requested epidural analgesia in active labor were randomly allocated into two groups; one group received 20 mL of ropivacaine 0.125% with fentanyl 50 µg and the other received 20 mL of bupivacaine 0.125% with fentanyl 50 µg. The efficacy of analgesia, adverse effects, and obstetric and neonatal outcomes of both groups were compared.

**Results:**

There were no differences between the two study groups in the measured obstetric and neonatal outcomes. The onset time, duration of analgesia, and sensory levels were similar between the groups. Visual analog pain scale scores did not differ between the groups before analgesia or at any of the subsequent evaluation periods.

**Conclusion:**

Both ropivacaine and bupivacaine provide equivalent labor analgesia with high maternal satisfaction and tolerable adverse effects in the clinically used dose range. No adverse obstetric or neonatal outcomes were observed in either group. Therefore, either drug is a reasonable choice for labor analgesia and can be used without jeopardizing the safety of the mother and fetus.

## PRECIS:

Ropivacaine and bupivacaine seem to be equiopotent at clinically used concentrations and can both be reasonable choices for labor analgesia.

## INTRODUCTION

Labor pain is reported to be one of the most severe pains that have ever been evaluated^(1,2)^. In a previous study, 41% of women considered it as the worst experience that they had ever had. Fear of labor pain seems one of the most important reasons for the tendency to cesarean section^(2)^. Additionally, pain-induced maternal sympathetic activation in labor compromises fetal oxygenation. Therefore, effective pain relief during labor is essential to reduce maternal and perinatal morbidity and to avoid unnecessary cesarean sections performed due to maternal anxiety^(3)^.

Walking epidural analgesia on labor reveals lower pain scores, leading to higher maternal satisfaction with better cardiovascular and pulmonary physiology^(3)^. The ideal drugs to be used for labor analgesia should have a long duration of action with minimum motor blockade, limited placental transfer, and no significant adverse effects on the mother and fetus^(4,5)^. Bupivacaine is the most commonly used drug for this purpose. Despite the extensive use and relative safety of bupivacaine, newer drugs such as ropivacaine and levobupivacaine have been developed as alternative agents to decrease the risk for cardiac and central nervous system toxicity. Another advantage of these drugs is less motor blockade compared with bupivacaine^(6)^. The addition of opioids to these local anesthetics such as sufentanil or fentanyl is preferable due to their dose minimizing and adverse-effect-reducing properties^(7)^.

The purpose of the current study was to compare the effects on obstetric and neonatal outcomes between ropivacaine and bupivacaine in combination with fentanyl used in walking epidural analgesia.

## MATERIALS AND METHODS

This prospective randomized controlled trial was conducted at a tertiary center during a one-year period. The study was approved by the Cerrahpaşa University Local Ethics Committee (approval number: P20/1999). Written consent for participation was obtained prior to recruitment into the study.

Women aged 18-35 years, classified as American Society of Anesthesiologists score I and II who requested epidural analgesia in active labor with cervical dilatation 3-4 cm, and uterine contractions ≥3/10 minutes between 37-41 weeks’ gestational age with a singleton pregnancy in the vertex position were enrolled in this study.

Women with high risk pregnancies as defined by the obstetrician such as severe preeclampsia, insulin-dependent diabetes mellitus, multiple pregnancies or with any contraindications to epidural techniques such as coagulopathies, spinal deformities, local infections, and any sensitivity to the drug were excluded.

The patients were randomized 1:1 to each treatment arm, with stratification based on parity. One hundred participants who met the above mentioned criteria were allocated into two groups. Group R received 20 mL of ropivacaine 0.125% with fentanyl 50 µg, and group B received 20 mL of bupivacaine 0.125% with fentanyl 50 µg.

No sedative premedication was given to the participants. After intravenous prehydration with 500 mL 0.09% NaCl solution, a 16-gauge Touhy needle was placed in the patients at the level of L3-4 or L4-5 interspaces via a midline approach under complete aseptic conditions. The loss of resistance technique was used to identify the epidural space. After monitoring any aspirate of blood or cerebrospinal fluid via the catheter, a 3 mL test dose of the study medication was administered. If there were no signs of an intravascular or intrathecal injection for the following 5 minutes, the remaining dose of the selected medication was administered. The catheter was inserted about 3-4 cm into the epidural space and securely fixed. After the insertion, patients were placed in the supine position with left uterine displacement.

Vital parameters of the mother such as heart rate, blood pressure, respiratory rate, and maternal saturation were recorded before and every 15 minutes after the injection. Onset of analgesia was evaluated as the time after injection until the first painless contraction occurred. The effectiveness of the epidural block was evaluated using a visual analog pain scale (VAS) (VAS: 0 to 10, with 0 being no pain and 10 being the worst imaginable pain). An additional dose of 5 mL of the analgesic solution was injected whenever the parturient had VAS ≥3 during labor. The sensory level was assessed using the pinprick method. Preservation of motor function was determined using the modified Bromage scale in both legs (0: no paralysis, full flexion of knees and feet, 1: inability to raise the extended leg and ability to move knees and feet; 2: inability to move knees but ability to move feet; 3: inability to flex ankle joints, complete motor blockade of lower limbs). Maternal adverse effects during the procedure such as nausea, vomiting, pruritus, bradycardia, trembling, and hypotension were recorded.

Fetal wellbeing and uterine contractions were monitored using cardiotochography. For the comparison of uterine activity, a 30-minute postinjection period was taken into account. The duration of the first and second stages of labor, and mode of delivery were recorded. Neonatal welfare was assessed using Apgar scores at 1 and 5 minutes. Maternal satisfaction about labor analgesia was determined after 24 hours on a four-point scale.

### Statistical Analysis

Data were analyzed using IBM SPSS 22.0 software (SPSS Inc., IBM, Chicago, Illinois, USA), and descriptive data are expressed as mean ± standard deviations and frequencies. The Mann-Whitney U test, Student’s t-test, and chi-square test were used for comparisons. A probability (p) value of  <0.05 was considered significant.

## RESULTS

The enrolled 100 women were assigned to either the ropivacaine group (group R) (n=50) or the bupivacaine group (group B) (n=50). The demographic characteristics were similar between the two groups. Maternal and fetal hemodynamic data were also comparable (Table 1).

Maternal adverse effects (nausea and pruritus) were seen in both groups (group B: 20%, group R: 10%; group B: 10%, group R: 20%, respectively). Trembling was only seen in two patients of group R. There were no cases of motor blockade in either group.

The onset time, duration of analgesia, and sensory levels were similar between the groups. VAS scores did not differ between the groups before analgesia or at any of the subsequent evaluation periods. Ten parturients in group R and 11 in group B required an additional bolus of 5 mL after 2-3 hours (Table 2).

Maternal satisfaction with labor analgesia was mostly defined as excellent in both groups and no significant difference was observed between the groups (Table 2).

Obstetric characteristics and outcomes are shown in Table 3. Four parturients in each group required cesarean section and one parturient required forceps application in group B. No significant difference was found between the groups when assessed for uterine activity.

Twenty percent of patients in group B and 28% in group R required local anesthesia for closure of the episiotomy wound.

There were no differences between the two study groups in the measured neonatal outcomes (Table 3).

## DISCUSSION

Epidural analgesia has become a widely-used technique for providing pain relief in labor. Nowadays, there is an increase in the number of the epidural drugs. The most recent literature focuses on new enantiomers such as ropivacaine, which have reduced risk of cardiotoxicity compared with bupivacaine^(7)^. In our comparison of these two agents in the present study, no motor blockade was observed and maternal satisfaction rates were similar with tolerable adverse effects. In addition, no obstetric or neonatal adverse effects were observed.

Some previous studies claimed that epidurals prolonged labor, and increased oxytocin requirements and instrumental and operative delivery rates^(8,9)^. This was explained as motor block in perineal and abdominal muscles caused by epidural local anesthetics, which may cause abnormal internal rotation of the fetal head leading to dystocia^(9)^.

In a meta-analysis, it was suggested that the type of epidural analgesia might influence spontaneous vaginal delivery rates. Analgesia combined with low-dose opioid and local anesthetic has been asserted to result in lower rates of instrumental deliveries^(10,11)^. Some investigators suggested that ropivacaine was associated with an increased rate of spontaneous vaginal delivery compared with bupivacaine due to a reduction in motor block^(12)^. Lv et al.^(7)^ reported in their meta-analysis of 10 impact studies that ropivacaine was associated with less motor blockade but a higher incidence of instrumental delivery. Halpern et al.^(13)^ showed that the rate of motor block was more frequent in the bupivacaine group but the incidence of spontaneous vaginal delivery was similar regardless of whether ropivacaine or bupivacaine were used for labor analgesia. There are conflicting results in the literature in the comparison of these two local anesthetics regarding the mode of delivery. In the current study, the vaginal spontaneous labor rate was high and there was no significant difference between the groups in regard to operative delivery.

It is assumed that ropivacaine has a greater selectivity for sensory fibers than motor fibers due to its lower lipophilic capacity compared with bupivacaine. Accordingly, it is less likely to cause motor blockade and neurotoxicity^(4,6)^. There were no cases of motor blockade in either group in our study. This could be related to the use of very low and titrated concentrations of a local anesthetic through the addition of opioids. It may also account for our high spontaneous vaginal delivery rate. Higher concentrations of local anesthetic may be the reason of increased motor blockade and instrumental delivery rates in previous studies.

Lee at al.^(14)^ reported that bupivacaine was associated with prolongation in the first stage of labor. This may result from higher concentrations of initiated analgesia with a 0.25% solution, which triggers motor block, leading to elongation of labor. In contrast, other comparative studies using these local anesthetics in a range of 0.075-0.125% found no differences in the durations of the first or second stages of labor, similar to our results^(15,16)^.

Our findings regarding neonatal outcomes were comparable with the literature^(4,13,14,15,16)^. There were no significant differences in the indicators of neonatal wellbeing between the two groups. In a study conducted by Writer et al.^(12)^, lower neurologic and adaptive capacity scores with bupivacaine versus ropivacaine were found. We did not assess this outcome due to the conflicting results about its reliability in newborn evaluations^(17)^.

Shokry et al.^(18)^ compared two groups receiving 0.125% bupivacaine and 0.2% ropivacaine, each with fentanyl 100 µg and found an non-significant faster onset of action and significantly shorter duration of analgesia in the ropivacaine group. In contrast, Chora and Hussain^(4)^ showed significantly faster onset of analgesia in the bupivacaine group and longer duration in the ropivacaine group. Unlike these, the onset and duration of analgesia for both groups was comparable in current study, consistent with the research of Beilin et al.^(19)^.

Bawdane et al.^(20)^ recorded similar pain scores, sensory levels, and overall maternal satisfaction between the two groups, as we observed. Although ropivacaine is suggested to be less potent than bupivacaine^(21)^, they appear to be equipotent at clinically used concentrations.

### Study Limitations

The limitation of the current study is its small sample size in both groups, further research should be organized with large sample groups.

## CONCLUSION

Overall, both ropivacaine and bupivacaine can provide equivalent labor analgesia with high maternal satisfaction and tolerable adverse effects in the clinically used dose range. A combination with opioids is preferable considering their dose lowering effect. No adverse obstetric or neonatal outcomes were observed in either group in the current study. Therefore, from a clinical perspective, either drug is a reasonable choice for labor analgesia and can be used without jeopardizing the safety of the mother and fetus.

## Figures and Tables

**Table 1:**
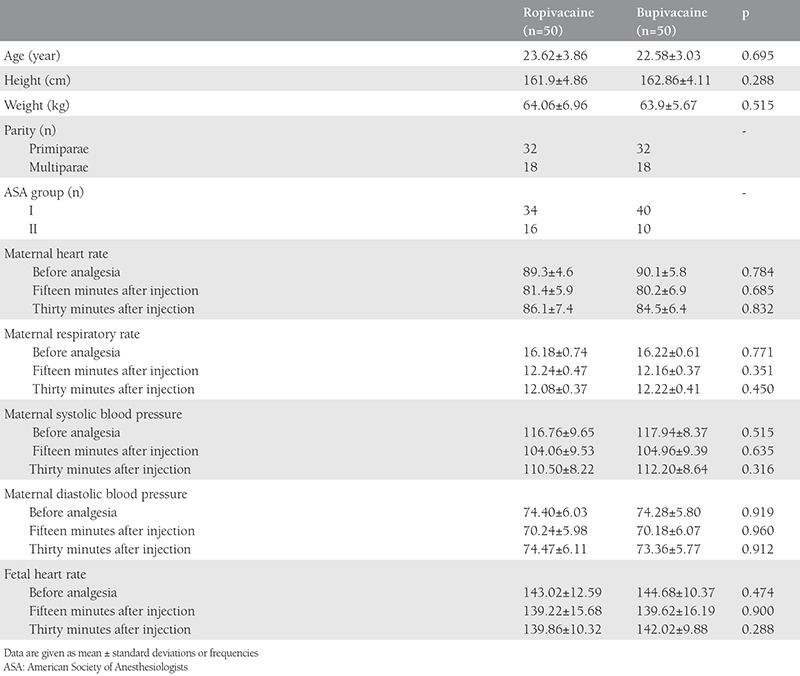
Demographic characteristics of the patients and data of maternal and fetal hemodynamic parameters

**Table 2:**
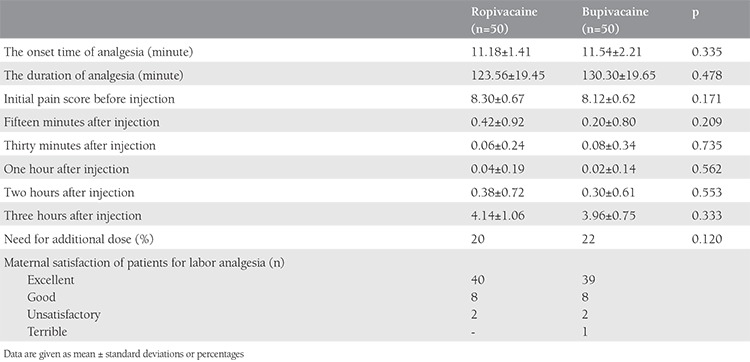
Effectiveness of analgesics in both groups and pain assessment using the 0-10 visual analogue scale

**Table 3:**
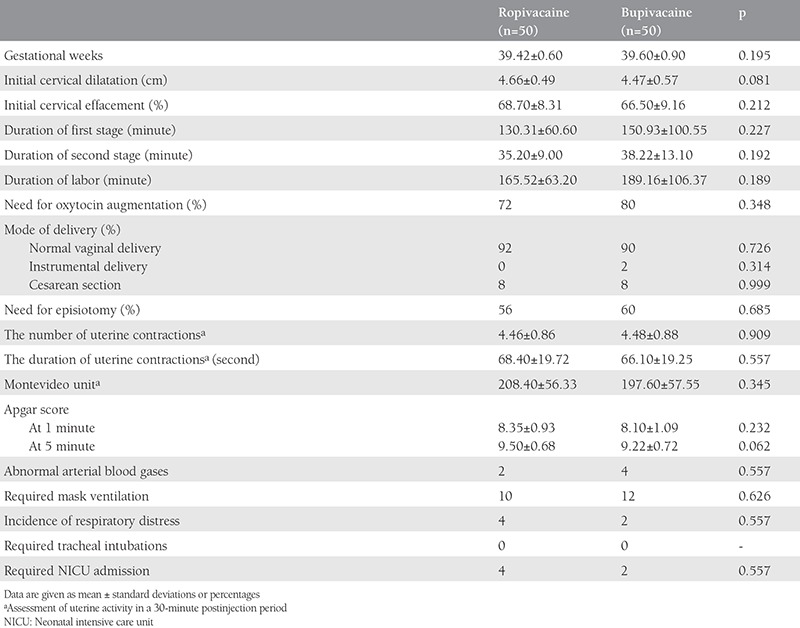
Obstetric characteristics and data of obstetric and neonatal outcomes
